# The Current State of 3D-Printed Prostheses Clinical Outcomes: A Systematic Review

**DOI:** 10.3390/jfb16100370

**Published:** 2025-10-01

**Authors:** Huthaifa Atallah, Titeana Qufabz, Rabee Naeem, Hadeel R. Bakhsh, Giorgio Ferriero, Dorottya Varga, Evelin Derkács, Bálint Molics

**Affiliations:** 1 Prosthetics and Orthotics Department, School of Rehabilitation Sciences, The University of Jordan, Amman 11942, Jordan; titeanaqofabz@hotmail.com; 2 Digital Fabrication Division, Revolutionary Technologies for Medical Solutions, Amman 11190, Jordan; rabeenaeem1@gmail.com; 3 Department of Rehabilitation Sciences, College of Health and Rehabilitation Sciences, Princess Nourah bint Abdulrahman University, Riyadh 11564, Saudi Arabia; hrbakhsh@pnu.edu.sa; 4 Department of Biotechnology and Life Sciences, University of Insubria, 21100 Varese, Italy; giorgio.ferriero@icsmaugeri.it; 5 Physical and Rehabilitation Medicine Unit, Scientific Institute of Tradate, Istituti Clinici Scientifici Maugeri IRCCS, Tradate, 21049 Varese, Italy; 6 Doctoral School of Health Sciences, Faculty of Health Sciences, University of Pécs, 7621 Pécs, Hungary; vargadorka96@gmail.com (D.V.); evelin.derkacs@etk.pte.hu (E.D.); 7 Department of Sport Physiotherapy, Faculty of Health Sciences, University of Pécs, 7621 Pécs, Hungary; molics.balint@etk.pte.hu

**Keywords:** 3D printing, systematic review, additive manufacturing, clinical outcomes, prosthetic socket

## Abstract

**Introduction**: 3D-printing is an emerging technology in the field of prosthetics, offering advantages such as cost-effectiveness, ease of customization, and improved accessibility. While previous reviews have focused on limited aspects, the aim of this systematic review is to provide a comprehensive evaluation of the clinical outcomes of 3D-printed prostheses for both upper and lower limbs. **Methods**: A search was conducted following Preferred Reporting Items for Systematic Reviews and Meta-Analyses (PRISMA) guidelines across six databases (PubMed, Web of Science, EBSCO, Scopus, Cochrane Library, and Sage). Studies on 3D-printed prostheses in human rehabilitation that focused on the clinical outcomes of the device were included, while studies lacking clinical data, 3D printing details, or focusing on traditional manufacturing methods were excluded. Finally, the risk of bias was assessed using the modified Downs & Black Checklist. **Results**: A total of 1420 studies were identified, with 11 meeting the inclusion criteria. The included studies assessed different 3D-printed prosthetic types and upper and lower limb prostheses. The main clinical outcomes analyzed were functional performance, design and material integrity, and overall effectiveness of 3D-printed prostheses. Studies on upper limb prostheses reported improved dexterity, range of motion (ROM), and user satisfaction, despite some durability limitations. Lower limb prostheses showed enhancements in comfort, gait parameters, and customization, particularly in amphibious and partial foot designs. **Conclusions**: 3D-printed prostheses show potential to improve functional performance, patient satisfaction, fit, and implementation feasibility compared to conventional methods. However, limitations such as small sample sizes, variability in assessment tools, and limited high-quality evidence highlight the need for further research to support broader clinical adoption.

## 1. Introduction

3D printing and scanning have emerged as promising technologies with the potential to transform the field of prosthetics [1,2]. These technologies offer a competitive alternative to conventional prosthetic manufacturing by providing advantages such as cost-effectiveness [3], customization [2], rapid reprinting [4,5], improved comfort [6], enhanced user satisfaction [7], better functional performance [8], higher acceptance among users [9], reduced fitting errors [10], and the possibility of remote fitting [11,12].

While previous systematic reviews have explored aspects of 3D-printed prostheses, they have been limited in scope. For example, Kim et al. [13] focused on the structural integrity of 3D-printed prosthetic sockets, Diment et al. [14] evaluated the clinical viability of upper-limb prostheses, and Abbady et al. [15] considered prosthetic applications in low- and lower–middle-income countries. However, to the best of the authors’ knowledge, no comprehensive review has systematically assessed the clinical outcomes of both upper and lower limb 3D-printed prostheses. Evaluating both types is important because upper and lower limb prostheses serve distinct functional roles—upper limb devices primarily support fine and gross motor activities, while lower limb devices are critical for mobility, gait, and weight-bearing function—yet both significantly impact patient quality of life, satisfaction, and independence.

This systematic review therefore aims to fill this gap by synthesizing the evidence on clinical outcomes, which are essential indicators of prosthetic effectiveness. The outcomes considered include functional performance (gait, strength, ROM, dexterity, physiological changes, Electromyography (EMG)), patient-reported outcomes (satisfaction, comfort, pain, quality of life, ease of use), design and material integrity, usage patterns and implementation feasibility, fit, and the overall effectiveness of 3D-printed prostheses. By providing a comprehensive evaluation, this review contributes new knowledge on how 3D-printed prosthetic devices impact patient function, experience, and clinical applicability, highlighting both opportunities and limitations for future clinical adoption.

## 2. Materials and Methods

### 2.1. Study Team

To ensure clinically robust and meaningful findings, the study was conducted by an inter-professional team comprising experts in evidence synthesis, quantitative research methods, prosthetics and orthotics, occupational therapy, and rehabilitation medicine.

### 2.2. Search Strategy and Data Sources

A systematic search was completed in April 2025 in compliance with Preferred Reporting Items for Systematic Reviews and Meta-Analyses (PRISMA) guidelines, and the study protocol was registered in PROSPERO (no. CRD420250611965). Data collection was carried out using six electronic databases: PubMed, Web of Science, EBSCO, Scopus, Cochrane Library, and Sage. To maximize coverage, reference lists of the included studies were also manually screened, and supplementary searches were performed in Google Scholar to capture any additional relevant publications. According to the Patient, Intervention, Comparison/control, Outcomes (PICO) framework, we used the following keywords (customized for each database): “3D Printing”, “3D Printed”, “Additive Manufacturing”, “Three-Dimensional Printing”, “Prostheses”, “Prosthetic”, “Prosthesis”, “Upper Limb”, “Upper Extremity”, “Lower Limb”, “Lower Extremity”. No language filters were used for the search strategy. The keywords applied in the search strategy are presented in Table S1.

We included papers assessing the clinical outcomes of both upper and lower limb 3D-printed prostheses, written in English language. Case reports, meta-analyses, systematic reviews, meeting abstracts, posters, and thesis papers were excluded.

### 2.3. Study Selection and Eligibility Criteria

Following the removal of duplicates, the retrieved titles and abstracts were organized in an Excel sheet for screening. Study selection was conducted in two phases. In the first phase, titles and abstracts were independently reviewed for eligibility, with any differences in judgment resolved through discussion. Assistance with data collection and literature review was also provided during this stage. In the second phase, the full texts of potentially eligible studies were assessed against the inclusion criteria outlined in Table 1, and any disagreements were settled through independent arbitration to maintain consistency in the selection process.

### 2.4. Data Extraction, Synthesis and Analysis

The following data items were extracted: first author and year of publication, country/setting, study design, sample size and population characteristics (e.g., age, amputation level), type of intervention (prosthetic design, material, or fabrication approach), comparison groups (if applicable), outcome measures (functional performance, patient-reported outcomes, design/material integrity, and feasibility), results, and authors’ main conclusions. Studies were included only when clinical outcomes showed sufficient consistency, with particular emphasis placed on functional performance (gait, strength, range of motion (ROM), dexterity, physiological changes, and Electromyography (EMG)), patient-reported outcomes (satisfaction, comfort, pain, quality of life (QoL), and ease of use), design and material integrity, usage patterns and implementation feasibility, fit, and overall effectiveness of 3D-printed prostheses. Clinical outcomes were grouped by prosthetic type, including upper limb prostheses and lower limb prostheses. Data extraction and cross-checking procedures were performed independently, and discrepancies were resolved through discussion to ensure accuracy. Data interpretation, synthesis, and quality review were conducted collaboratively. Data management was carried out using Excel and Word.

### 2.5. Methodological Quality Assessment

The quality of the selected articles was evaluated using the modified Downs & Black Checklist [16]. This tool was selected for its established validity in assessing the methodological rigor of both randomized and non-randomized controlled trials and is widely recognized as a robust instrument for quality appraisal in systematic reviews. In cases where consensus was not initially achieved, a final decision was reached through further review. The checklist consists of 27 items divided into five categories: study quality, external validity, internal validity bias, confounding selection bias, and study power, with a maximum score of 28 points. Each checklist item was allocated a score of one, with the exception of item 27, which carried two points. Based on the total scores, studies were classified into quality categories using established thresholds: poor (<14), fair (15–19), good (20–25), and excellent (26–28).

## 3. Results

**Figure 1:**
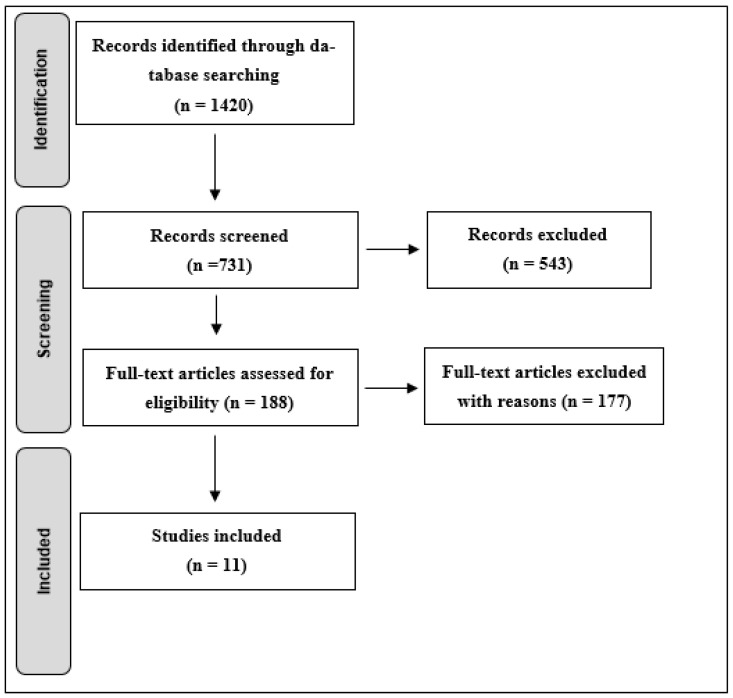
PRISMA (Preferred Reporting Items for Systematic Reviews and Meta-Analyses) flowchart.

A PRISMA flowchart of the study identification process is presented in Figure 1. Of the 1420 potential records, 731 studies underwent title/abstract screening, 188 were reviewed in full, and 11 studies were ultimately included for appraisal. Across the studies included, 7 reported improvements in dexterity, 5 noted increased comfort and satisfaction, 3 described enhanced range of motion, and 4 highlighted durability issues such as component breakage. Two lower-limb studies reported improved swimming performance and higher comfort with digitally designed sockets.

The search resulted in 1 cross-sectional study, 3 prospective cohort studies, and 7 studies employing other designs (such as comparative, qualitative, prospective observational, general prospective, descriptive, pilot, and feasibility studies). Complete details of the study characteristics are summarized in Table S2. Studies represented data from 177 participants across several countries, including the USA, Nepal, India, Korea, and Canada. Upper limb studies primarily focused on body-powered and myoelectric prostheses (Table 1). Improvements in gross manual dexterity and daily functional use were frequently reported, although fine motor tasks and device durability remained common limitations [3,8,9,11,17,18,19,20,21]. Lower limb studies demonstrated benefits in comfort, socket fit, and amphibious use (Table 2), though evidence remains limited to feasibility and pilot studies [6,22] (Table S3).

**Table 1:** 3D-printed upper limb prostheses.

Author	Year	Aim	Methods	Results	Conclusion
Shrestha and Gautam [9]	2023	To explore the functionality of 3D-printed prosthetic hands with respect to performance, usability, and user satisfaction.	Participants: 76 individuals (61 with trans-radial amputations and 15 with partial hand amputations) Device: 3D-printed prosthetic hands Tools: Semi-structured questionnaires, the Southampton Hand Assessment Procedure (SHAP), and follow-up interviews Measures: User satisfaction at three months, functional task performance, and prosthesis durability	Participants were able to lift only light objects. Participants had trouble with tasks requiring complex movements or lifting heavy objects. Only 47 participants (approximately 60%) reported complete satisfaction. The primary reasons for dissatisfaction were torn rubber bands or cords and broken components.	Participants were able to complete certain tasks using the 3D-printed prosthetic hands.
Belter et al. [8]	2016	To evaluate the effectiveness of a low-cost, 3D-printed body-powered prosthetic hand.	Participants: 12 able-bodied individuals and 2 individuals with trans-radial amputations Device: Multigrasp 3D-printed prosthetic hand Tools: Box and Blocks Test (BBT) apparatus, Southampton Hand Assessment Protocol (SHAP), load cells for force measurement, linear potentiometers for excursion data, and user feedback questionnaires Measures: Performance scores from the BBT and SHAP, force data during grasping, user-reported device preferences, and calculated Index of Function (IoF) and Functionality Profile (FP) scores Methods: Comparative analysis of three terminal devices: the multigrasp 3D-printed hand, a metal mechanical hook, and a metal mechanical hand	Able-bodied participants performed better with the 3D-printed hand compared to the other terminal devices (mechanical hand and hook). Participants with trans-radial amputations scored significantly higher with the hook than with the 3D-printed hand. Participants achieved the highest scores with the baseline (natural hand), followed by the mechanical hand, 3D-printed hand, and hook, in that order. Participants scored higher with the hook than with the 3D-printed hand.	The multigrasp 3D-printed prosthetic hand demonstrated comparable performance to existing terminal devices among inexperienced users. However, participants with trans-radial amputations scored lower with the 3D-printed hand compared to the mechanical hook. The availability of multiple grasp types, despite experiencing challenges with grip regulation. Overall, the 3D-printed hand shows promise in enhancing prosthetic functionality.
Zuniga et al. [17]	2016	Identify the effects of using a wrist-driven 3D-printed prosthetic hand over six months in children with upper-limb deficiencies.	Participants: 5 children with unilateral carpal-level upper-limb reductions (missing some or all fingers) Device: Body-powered 3D-printed prosthetic hand designed for pediatric use Tools: Gulick anthropometric tape measure, Lange skinfold caliper, goniometer, muscle testing dynamometer, and a study-specific short survey Measures: Anthropometric data, range of motion (RoM), muscular strength, prosthesis usage, and activity levels	Improvements were observed in forearm circumference and ROM for flexion and extension in the affected hand after six months. No significant changes were found in wrist flexion and extension strength, active ROM for ulnar and radial deviation, or forearm skinfold thickness. The 3D-printed prosthetic hand was used across a variety of activities, including recreational play, household tasks, school activities, and sports. Four children reported using the device for 1–2 h per day, while one child reported using it for 4–6 h daily.	Despite limitations in durability, environmental impact, and regulatory standards, 3D-printed prosthetic hands—such as the Cyborg Beast—offer an affordable and practical solution to enhance mobility for individuals with upper-limb deficiencies, particularly in developing countries.
Zuniga et al. [11]	2019	To describe the remote fitting process for 3D-printed upper-limb prostheses and evaluate patient satisfaction and comfort.	Participants: 8 individuals (6 with partial hand amputations and 2 with trans-radial amputations) Devices: Wrist-driven 3D-printed partial hand prosthesis and elbow-driven 3D-printed trans-radial prosthesis Tools: Quebec User Evaluation of Satisfaction with Assistive Technology (QUEST 2.0), Orthotics and Prosthetics Users Survey (OPUS), a study-specific general survey (assessing activity types, daily usage, comfort rating, impact on quality of life/self-esteem, and device malfunctions), and content and score analysis Measures: Patient satisfaction, comfort, perceived functional ability, rated ease or difficulty of specific tasks, OPUS Upper Extremity Functional Status scores, prosthesis usage, device integrity, and perceived impact	Participants reported high satisfaction with their 3D-printed prostheses, particularly regarding weight, safety, and ease of use. Tasks such as donning and doffing the prosthesis and drinking from a paper cup were perceived as the easiest to perform.	The remote fitting method for 3D-printed upper-limb prostheses shows promise for rapidly delivering functional devices in developing countries, leveraging widespread access to digital technologies even in rural areas.
Zuniga et al. [18]	2019	Identify the functional and strength changes associated with the use of 3D-printed body-powered upper-limb prostheses	Participants: 11 individuals (9 with partial hand amputations and 2 with trans-radial amputations) Device: 3D-printed, wrist-driven or elbow-driven body-powered prostheses Assessment Tools: Box and Block Test, strength testing dynamometer, anthropometric measurement tools, and a short survey Measured Outcomes: Gross manual dexterity, wrist strength, prosthesis usage, quality of life impact, and device durability/malfunction	A significant improvement in dexterity of the affected hand was observed following the intervention. Average wrist flexor strength increased, though the change was not statistically significant. Most children used the prosthesis for approximately two hours per day. Some children experienced device breakage or malfunction.	Use of a 3D-printed body-powered upper-limb prosthesis improves gross manual dexterity in children with upper-limb differences after several weeks of use. Despite some durability issues, the device remains a functional and practical body-powered option.
Bhat et al. [19]	2021	To assess the utility of 3D-printed upper-limb prostheses in children with congenital hand amputations, and to evaluate their functionality and cost-effectiveness.	Participants: 14 children (11 with partial hand amputations and 3 with trans-radial amputations) Device: 3D-printed wrist-driven and elbow-driven prostheses Assessment Tools: Unilateral Below Elbow Test (UBET), Box and Block Test, and ABILHAND questionnaire Measured Outcomes: Gross manual dexterity, upper-limb function, perceived ease of performing daily activities, performance comparisons, and differences by amputation level (specifically highlighting below-elbow cases)	A significant improvement in manual gross dexterity was observed when using the prosthesis. A significant decrease in performance scores was noted with the 3D-printed prosthesis on tasks likely requiring lateral or tripod pinch—functions the device lacked. Perceived difficulty increased significantly when using the 3D-printed prosthesis, further suggesting limitations due to the absence of specific pinch functionalities. Participants with trans-radial amputations performed better with the prosthesis compared to those with partial hand differences. Eleven out of fourteen participants completed the six-month follow-up.	3D-printed upper-limb prostheses improve gross grasping in children with congenital hand amputations but lack fine motor functions such as lateral and tripod pinch. Despite these limitations, they are affordable, easily replaceable, and serve effectively as transitional devices for growing children.
Ku et al. [20]	2019	Evaluate the clinical impact of a low-cost, 3D-printed myoelectric prosthetic hand with a user interface on patients’ daily lives	Participants: 10 individuals with trans-radial amputations. Device: Single-channel, myoelectric-interface, 3D-printed hand prosthesis Assessment Tools: OPUS-UEFS (Orthotics and Prosthetics Users Survey—Upper Extremity Functional Scale) and Visual Analog Scale (VAS) Outcome Measures: Upper extremity functionality (ability to perform 28 distinct activities) and changes in pain intensity following prosthetic application	After using the 3D-printed, single-channel myoelectric prosthesis for 3 months: Upper extremity function showed a significant improvement. Pain scores decreased slightly, but the change was not statistically significant.	The low-cost, 3D-printed prosthetic hand with a single-channel myoelectric interface shows potential to improve the quality of life of amputees through daily use.
Zuniga et al. [3]	2015	Describe a low-cost, 3D-printed prosthetic hand for children, and propose a remote fitting method to improve access to affordable prosthetic care	Participants: 11 individuals with partial hand amputations Device: 3D-printed prosthetic hand Tools: Photographs (with ruler), image editing software (Image J, version 1.46, NIH), 3D modeling software (Blender 7.2, Blender Foundation, Amsterdam, Netherlands), scaling software (MakerWare software, Makerbot Industries, Brooklyn, NY), a custom sizing chart (age vs. scale percentage), standard tape measure, goniometer, and a short, custom, non-validated survey Measures: Accuracy of distance measurements (photograph-based vs. direct), anthropometric data and wrist range of motion, agreement and bias between photo-based and direct measurement methods, performance of the age-based sizing chart, perceived impact on quality of life, and daily usage and activity levels.	No significant differences were found between photograph-based and direct measurements. Anthropometric measurements showed no major bias and demonstrated good agreement between methods. Wrist range of motion showed a small bias with a wider range of agreement. Survey results indicated a potential positive impact on quality of life and daily activities. Prosthesis usage averaged 1–2 h per day after 1–3 months, primarily during home activities, play, and for recreational use. The age-to-scale sizing chart provided an excellent fit across participants.	The low-cost, 3D-printed hand, combined with the proposed distance-fitting method, presents a promising and affordable prosthetic solution for underserved children.
Zuniga et al. [21]	2018	Assess using a wrist-driven, 3D-printed partial hand prosthesis for 6 months affects muscle coordination (co-activation index) in children with one-sided upper-limb reduction.	Participants: 9 individuals with congenital partial hand deficiencies or amputations. Device: Wrist-driven, 3D-printed transitional hand prosthesis. Tools: Surface electromyography (EMG) system with bipolar electrodes, muscle testing dynamometer, and a custom (non-validated) survey. Outcome Measures: Maximal isometric wrist strength, EMG amplitude, co-activation index, daily prosthesis usage (hours), and activities performed with the prosthesis. Comparisons: Affected vs. non-affected side and baseline vs. 6-month follow-up.	The affected hand exhibited greater co-activation, with wrist flexors being stronger than extensors. Six-Month Follow-Up Comparisons (Before vs. After): Co-activation during wrist flexion decreased significantly over time. The affected hand showed a substantially greater reduction in co-activation compared to the non-affected hand. The non-affected hand remained stronger during wrist extension. No significant changes in wrist strength were observed over the six-month period. Average daily prosthesis use was approximately a few hours, primarily for recreational activities such as play and fun. Only a subset of participants completed the six-month follow-up assessment.	The wrist-driven, 3D-printed hand prosthesis significantly reduced the co-activation index in children with congenital upper-limb reduction deficiencies, potentially enhancing motor control strategies and improving outcomes in prosthetic rehabilitation.

**Table 2:** 3D-printed lower limb prostheses.

Author	Year	Aim	Methods	Results	Conclusion
Goldstein et al. [6]	2020	Assess the usability and acceptance of a 3D-printed amphibious prosthesis and compare its performance to that of a standard Swim Ankle.	Participants: 10 individuals with trans-tibial amputations. Device: A 3D-printed amphibious lower-limb prosthesis designed for swimming and seamless transition between land and water environments. Comparison: Performance and usability of the 3D-printed amphibious prosthesis were compared to those of a standard Swim Ankle prosthesis. Tools: Customized Likert-scale survey (assessing likability, ease of use, comfort, balance, energy expenditure, pain, swimming quality, satisfaction, and user preferences), stopwatch/timer, automated blood pressure monitor, verbal and written feedback collection, and clinical inspection of residual limb skin integrity (e.g., redness, breakdown) Outcome Measures: Subjective assessments survey (likability, ease of use, comfort, energy demands, balance, pain, satisfaction, and overall preferences), performance-based assessments (Time to complete typical pool tasks (e.g., water entry and exit, 25m lap swim, treading water)), and physiological measures (Changes in vital signs and calculated Rate Pressure Product (RPP)).	The novel 3D-printed amphibious prosthesis significantly outperformed the standard Swim Ankle in pool entry and exit tasks using both the ladder and ramp. Lap swim times and changes in vital signs were comparable between the two devices. Users preferred the new prosthesis for its lighter weight, greater comfort, and overall satisfaction, reporting it to be more secure and convenient.	The novel 3D-printed amphibious lower-limb prosthesis was well-received and demonstrated good user-friendliness among a small group of participants in a recreational setting. These positive findings suggest potential benefits of the device for future use.
Eshraghi et al. [22]	2024	To explore the feasibility of a protocol for analyzing geometric and clinical differences between manually designed and digitally designed 3D-printed prosthetic sockets for individuals with trans-tibial amputations, with the aim of informing a future larger trial.	Participants: 9 individuals with trans-tibial amputations. Device: Each participant received two 3D-printed prosthetic sockets: one fabricated from a manually modified cast and one from a digitally modified scan. Comparison: The manually designed socket was compared to the digitally designed socket within each participant. Tools: 3D scanner, 3D modeling/CAD software, Socket Comfort Score, and a patient experience survey. Measures: Feasibility metrics (recruitment, drop-out, protocol deviations, adverse events), procedural times (casting, scanning, modification, printing), socket geometries, Socket Comfort Scores, and patient-reported experience and preference.	One participant deviated from the protocol, and one adverse event (a cracked socket) was reported. Casting took longer than scanning; however, digital modifications required more time. Eight out of nine participants rated the digital socket as comfortable. Manual sockets exhibited greater vertical height and larger volume compared to digital sockets. Participants preferred 3D scanning over manual casting.	3D scanning and digital design enhance socket production and patient engagement but face challenges such as limited tactile feedback, post-processing requirements, and variability among 3D printers. Overcoming regulatory and practical barriers, along with advances in materials and technology, will facilitate wider adoption in prosthetics and orthotics.

The Modified Downs & Black checklist ratings of included studies are summarized in Table S4. One of the 11 studies included in this systematic review was classified as poor quality, 9 were classified as fair, and one was classified as Good. Given that the majority of studies were rated as fair quality, with only one study classified as good, findings should be interpreted cautiously. The limited methodological rigor reduces the strength and generalizability of the evidence.

## 4. Discussion

This review examines the clinical outcomes of 3D-printed prostheses, including both upper and lower limb designs, with a focus on functional performance (gait, strength, ROM, dexterity, physiological changes, EMG), patient-reported outcomes (satisfaction, comfort, pain, QoL, ease of use), design and material integrity, usage patterns, implementation feasibility, fit, and overall effectiveness. Across studies, 3D printing has demonstrated the potential to improve accessibility, personalization, and patient satisfaction, though limitations in device durability and fine-motor performance remain common.

Previous systematic reviews have addressed certain aspects of 3D-printed prostheses. Kim et al. [13] focused on the structural integrity of transtibial sockets, Diment et al. [14] examined upper-limb prostheses but highlighted a lack of randomized controlled trials, and Abbady et al. [15] reviewed applications in low- and lower–middle-income countries. However, none of these reviews comprehensively evaluated clinical outcomes across both upper and lower limb devices, nor did they integrate functional, patient-reported, and implementation-related outcomes. By synthesizing evidence from multiple prosthetic types, our review provides a broader understanding of real-world effectiveness and user experience.

### 4.1. 3D-Printed Upper Limb Prostheses

Across the reviewed literature, 3D-printed upper limb prostheses consistently improved gross grasp, manual dexterity, and user satisfaction. Limitations in fine-motor tasks and device durability were frequently reported, particularly for body-powered designs. Myoelectric devices showed promise in enhancing functional performance, though evidence remains limited [20]. Our review expands upon Diment et al.’s work [14] by including more recent studies, reporting additional patient-centered outcomes such as ease of use, comfort, daily usage, and neuromuscular adaptations [3,8,9,11,17,18,19,20,21]. Studies also indicate that remote fitting and digital sizing techniques, including photographic measurements, can improve accessibility and user comfort without compromising fit [3,11,17,18]. Overall, the pattern suggests that 3D-printed upper limb prostheses enhance gross motor function and engagement in daily activities, but durability and fine-motor precision remain barriers to widespread clinical adoption.

### 4.2. 3D-Printed Lower Limb Prostheses

3D-printed lower limb prostheses demonstrate clear advantages in comfort, fit, and specialized functionality, including amphibious designs for recreational use [6]. Digital socket workflows generally improved efficiency and user experience, though technical and regulatory barriers remain [22]. While Kim et al. [13] focused solely on socket integrity, our review evaluates clinical outcomes such as gait, comfort, satisfaction, and implementation feasibility, offering a more comprehensive assessment. Across studies, improvements in gait biomechanics, patient satisfaction, and device usability were consistently observed. This suggests that additive manufacturing can provide versatile solutions for lower limb prostheses, though broader clinical adoption will require continued refinement of digital design workflows and material durability.

Overall, while the findings of this review highlight the potential of 3D-printed prostheses, it is important to emphasize the limited quality and scope of the current evidence. Most available studies are small in scale, lack rigorous designs such as randomized controlled trials, and employ heterogeneous outcome measures. As such, caution is warranted when interpreting these results. At present, there is insufficient high-quality scientific evidence to support broad conclusions regarding the efficacy, accessibility, or equity of 3D-printed prostheses. Further well-designed clinical studies are required before these technologies can be considered established alternatives to conventional prosthetic manufacturing.

### 4.3. Study Limitation and Future Recommendation

This review has several limitations. Many included studies were case series with small sample sizes, limiting generalizability. Heterogeneity in assessment methods, including variable questionnaires for patient satisfaction, made comparisons across studies difficult. No studies were identified on 3D printing for other common prosthetic types, such as above-knee devices. Finally, only one study met criteria for high methodological quality. Future research should focus on standardized evaluation protocols, long-term durability, and broader applications across diverse prosthetic types to strengthen the evidence base and facilitate clinical adoption. By addressing these gaps, future studies can build on our findings and expand the practical clinical impact of 3D-printed prosthetic technologies.

## 5. Conclusions

The reviewed evidence suggests that 3D printing holds promise for the development of both upper and lower limb prostheses, particularly with respect to personalization, accessibility, patient satisfaction, and certain aspects of functional performance. However, the findings should be interpreted with caution, as most included studies were of limited methodological quality, small in scale, and heterogeneous in outcome measures, with only one study rated as “Good.” Current evidence does not yet provide sufficient scientific support to draw firm conclusions about the overall efficacy, accessibility, or equity of 3D-printed prostheses compared to conventional approaches. Future research should prioritize well-designed clinical trials with larger sample sizes, standardized outcome measures, and long-term follow-up to more rigorously evaluate both the benefits and limitations of 3D-printed prosthetic technologies and to guide their safe and effective integration into clinical practice.

## Data Availability

No new data were created or analyzed in this study. Data sharing is not applicable to this article.

## Supplementary Materials

The following supporting information can be downloaded at: https://www.mdpi.com/article/10.3390/jfb16100370/s1, Table S1: Database Search Strategy and Keywords; Table S2: Characteristics of included studies; Table S3: Clinical outcomes; Table S4: Bias Assessment Scores and Levels.

## Abbreviations

The following abbreviations are used in this manuscript:

PRISMA	Preferred Reporting Items for Systematic Reviews and Meta-Analyses
ROM	Range of Motion
EMG	Electromyography
QoL	Quality of Life
