# Supplementary material for: The Current State of 3D-Printed Prostheses Clinical Outcomes: A Systematic Review
Source: J Funct Biomater. 2025 Oct 1;16(10):370. doi: 10.3390/jfb16100370 (PMC12565071; doi:10.3390/jfb16100370)
Supplement: Supplementary file 1 [file jfb-16-00370-s001.zip › jfb-3897697-supplementary/Table S1.pdf]

Table S1: Database Search Strategy and Keywords.

Database	Search strategy and keywords		Criteria of studies
**PubMed**	("3D Printing" OR "3D Printed" OR "Additive Manufacturing" OR "Three-Dimensional Printing") AND (("Prostheses" OR "Prosthetic" OR "Prosthesis") AND ("Upper Limb" OR "Upper Extremity" OR "Lower Limb" OR "Lower Extremity"))	**Inclusion**	• English language<br>• Cross-sectional, Randomized Controlled trials, and Prospective cohort studies<br>• Involving human subjects<br>• Investigated and reported outcomes of 3D-printed prosthetics for upper and lower limbs<br>• Reported clinical outcomes: gait parameters, comfort, usage, fit, effectiveness, or/and patient satisfaction.
**Web of Science**	("3D Printing" OR "3D Printed" OR "Additive Manufacturing" OR "Three-Dimensional Printing") AND (("Prostheses" OR "Prosthetic" OR "Prosthesis") AND ("Upper Limb" OR "Upper Extremity" OR "Lower Limb" OR "Lower Extremity"))		

**EBSCO**	("3D Printing" OR "3D Printed" OR "Additive Manufacturing" OR "Three-Dimensional Printing") AND (("Prostheses" OR "Prosthetic" OR "Prosthesis") AND ("Upper Limb" OR "Upper Extremity" OR "Lower Limb" OR "Lower Extremity"))		
**Scopus**	("3D Printing" OR "3D Printed" OR "Additive Manufacturing" OR "Three-Dimensional Printing") AND (("Prostheses" OR "Prosthetic" OR "Prosthesis") AND ("Upper Limb" OR "Upper Extremity" OR "Lower Limb" OR "Lower Extremity"))	**Exclusion**	• Focused on non-human subjects (e.g., biomechanical models)<br>• No clinical data or patient-related outcomes<br>• Lacked sufficient detail on the 3D-printing process, design, and production<br>• Examined only the printing process or materials without fitting on human subjects<br>• Reporting on traditional (non-3D-printed) prostheses<br>• Publication in other languages<br>• Case reports, Meta-analyses, Systematic reviews, Meeting abstracts, posters, and thesis papers.

**Cochrane Library**	("3D Printing" OR "3D Printed" OR "Additive Manufacturing" OR "Three-Dimensional Printing") AND (("Prostheses" OR "Prosthetic" OR "Prosthesis") AND ("Upper Limb" OR "Upper Extremity" OR "Lower Limb" OR "Lower Extremity"))		
**Sage**	("3D Printing" OR "3D Printed" OR "Additive Manufacturing" OR "Three-Dimensional Printing") AND (("Prostheses" OR "Prosthetic" OR "Prosthesis") AND ("Upper Limb" OR "Upper Extremity" OR "Lower Limb" OR "Lower Extremity"))		
