# Supplementary material for: The Current State of 3D-Printed Prostheses Clinical Outcomes: A Systematic Review
Source: J Funct Biomater. 2025 Oct 1;16(10):370. doi: 10.3390/jfb16100370 (PMC12565071; doi:10.3390/jfb16100370)
Supplement: Supplementary file 1 [file jfb-16-00370-s001.zip › jfb-3897697-supplementary/Table S2.pdf]

**Table S2: Characteristics of included studies.**

| | Author | Sample Size | Country | Study Design |
|---|---|---|---|---|
| **Upper Limb Prostheses** | Shrestha and Gautam (2023) (9) | 76 | Nepal | Cross-Sectional |
| | Belter et al. (2016) (8) | 14 | USA | Comparative Study |
| | Zuniga et al. (2016) (16) | 5 | USA | Prospective Cohort Study |
| | Zuniga et al. (2019) (11) | 8 | USA | Qualitative Study |
| | Zuniga et al. (2019) (17) | 11 | USA | Prospective Cohort Study |
| | Bhat et al. (2021) (18) | 14 | India | Prospective Observational Study |
| | Ku et al. (2019) (19) | 10 | Korea | Prospective Study |
| | Zuniga et al. (2015) (3) | 11 | USA | Descriptive Study |
| | Zuniga et al. (2018) (20) | 9 | USA | Prospective Cohort Study |
| **Lower Limb Prostheses** | Goldstein et al. (2020) (6) | 10 | USA | Pilot Study |
| | Eshraghi et al. (2024) (21) | 9 | Canada | Feasibility Study |
