# Supplementary material for: The Current State of 3D-Printed Prostheses Clinical Outcomes: A Systematic Review
Source: J Funct Biomater. 2025 Oct 1;16(10):370. doi: 10.3390/jfb16100370 (PMC12565071; doi:10.3390/jfb16100370)
Supplement: Supplementary file 1 [file jfb-16-00370-s001.zip › jfb-3897697-supplementary/Table S3.pdf]

Table S3: Clinical outcomes.

Type	Author	Clinical Outcomes					
		Functional Performance (Gait, strength, ROM, dexterity, physiological changes, and EMG)	Patient-Reported Outcomes (Satisfaction, comfort, pain, QoL, and ease of use)	Design and material integrity	Usage patterns and implementation feasibility	Fit	Effectiveness
Upper limb prostheses	Shrestha and Gautam (2023) (9)	X	X	X			X
	Belter et al. (2016) (8)	X	X				X
	Zuniga et al. (2016) (16)	X			X	X	X
	Zuniga et al. (2019) (11)	X	X	X	X		X
	Zuniga et al. (2019) (17)	X	X	X	X		X
	Bhat et al. (2021) (18)	X	X				X
	Ku et al. (2019) (19)	X	X				X
	Zuniga et al. (2015) (3)	X	X		X	X	X
	Zuniga et al. (2018) (20)	X			X		X
Lower limb prostheses	Goldstein et al. (2020) (6)	X	X			X	X
	Eshraghi et al. (2024) (21)		X	X	X	X	X
