# Supplementary material for: The Current State of 3D-Printed Prostheses Clinical Outcomes: A Systematic Review
Source: J Funct Biomater. 2025 Oct 1;16(10):370. doi: 10.3390/jfb16100370 (PMC12565071; doi:10.3390/jfb16100370)
Supplement: Supplementary file 1 [file jfb-16-00370-s001.zip › jfb-3897697-supplementary/Table S4.pdf]

Table S4: Bias Assessment Scores and Levels.

Orthotic type	Author	Score	Level
Upper Limb Prostheses	Shrestha and Gautam (2023) (9)	15	**Fair**
	Belter et al. (2016) (8)	17	**Fair**
	Zuniga et al. (2016) (16)	15	**Fair**
	Zuniga et al. (2019) (11)	14	**Poor**
	Zuniga et al. (2019) (17)	15	**Fair**
	Bhat et al. (2021) (18)	16	**Fair**
	Ku et al. (2019) (19)	16	**Fair**
	Zuniga et al. (2015) (3)	15	**Fair**
	Zuniga et al. (2018) (20)	16	**Fair**
Lower Limb Prostheses	Goldstein et al. (2020) (6)	19	**Fair**
	Eshraghi et al. (2024) (21)	21	**Good**
